# Supplementary material for: Symmetrically pulsating bubbles swim in an anisotropic fluid by nematodynamics
Source: Nat Commun. 2024 Feb 9;15:1220. doi: 10.1038/s41467-024-45597-1 (PMC10858235; doi:10.1038/s41467-024-45597-1)
Supplement: Supplementary file 1 — Supplementary Information [file 41467_2024_45597_MOESM1_ESM.pdf]

Supplementary Information: Symmetrically pulsating bubbles swim in an anisotropic fluid by nematodynamics

Sung-Jo Kim[1,2], Žiga Kos[3,4,5], Eujin Um[1], and Joonwoo Jeong[1*]

[1]Department of Physics, Ulsan National Institute of Science and Technology, Ulsan, Republic of Korea

[2]Center for Soft and Living Matter, Institute for Basic Science, Ulsan, Republic of Korea

[3]Faculty of Mathematics and Physics, University of Ljubljana, Ljubljana, Slovenia

[4]Jožef Stefan Institute, Ljubljana, Slovenia

[5]International Institute for Sustainability with Knotted Chiral Meta Matter, Hiroshima University, Higashihiroshima, Japan

[*]jjeong@unist.ac.kr

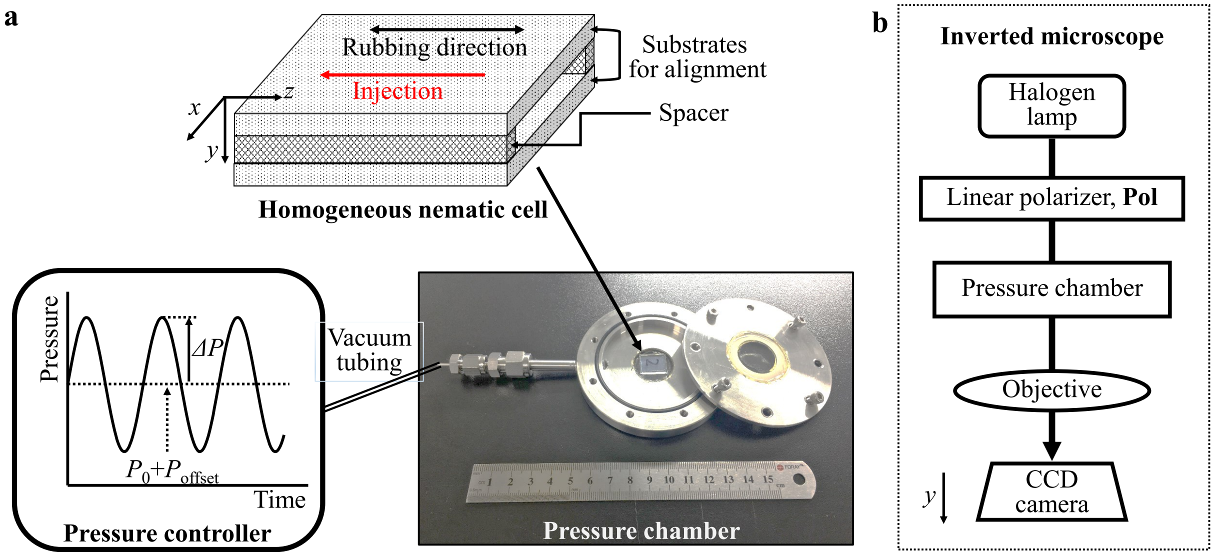

Supplementary Fig. 1. Experimental setup. **a**, Pressure modulating system to modulate the size of bubbles dispersed in the NLC. Bubble-dispersed NLC is injected into a homogeneous nematic cell with rubbed substrates. The cell is placed in a pressure chamber under controlled pressure. **b** Optical microscopy to observe pulsating bubbles.

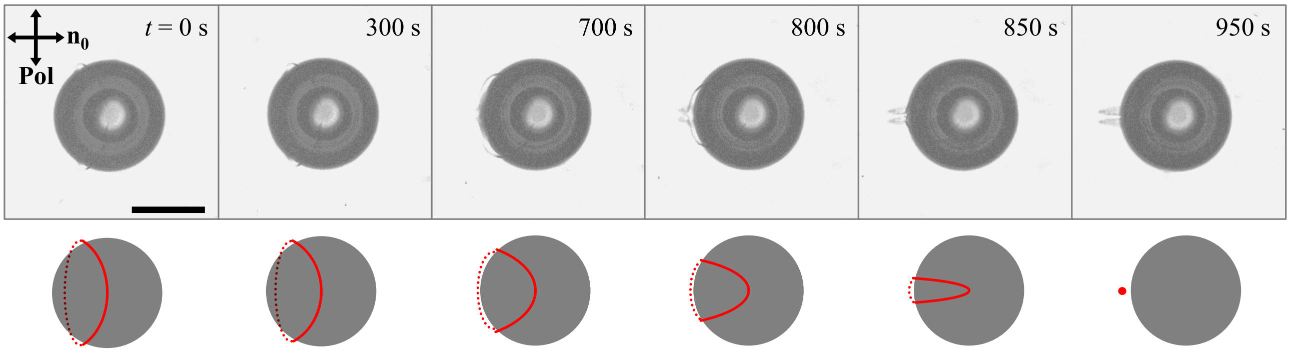

Supplementary Fig. 2. Transformation of SR into HH. The images in the top row are polarised optical microscopy images after adjusting the contrast and brightness to enhance visualisation of the disclination on the bubble surface. The bottom row illustrates the transformation process, the red solid line indicates the SR, and the dotted line represents the hidden part of the SR behind the bubble. At 950 s, the shrunken SR becomes the HH, represented by a red dot. The scale bar is 50 μm.

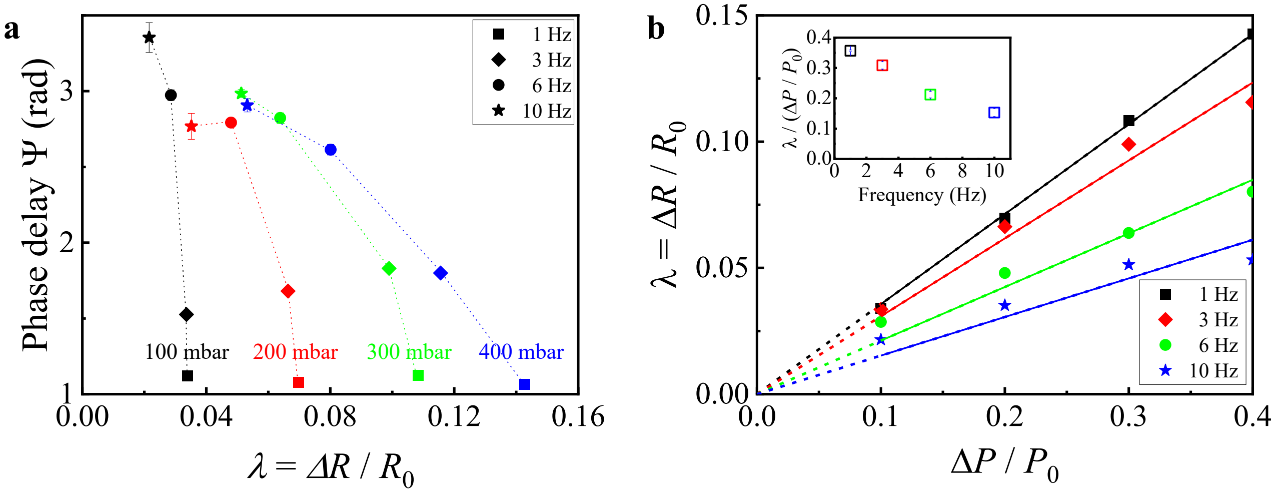

Supplementary Fig. 3. The relations among phase delay, pulsation ratio, and pressure modulation amplitude. **a**. Phase delay Ψ regarding pulsation ratio $\lambda = \frac{\Delta R}{R_0}$. Each symbol in the graph represents a different pulsation frequency, and a different color corresponds to a different pressure modulation amplitude ΔP ranging between 100 and 400 mbar. We show only representative data here with standard deviation as error bars; most error bars are smaller than symbols. While the phase delay decreases as we decrease the frequency, we find no clear correlation between the phase delay and λ; For example, Φ changes irregularly at 10 Hz, but is almost constant regardless of λ at 1 Hz. **b**. Pulsation ratio λ versus pressure-modulation amplitude ΔP. Error bars are smaller than symbols. As ΔP increases from 100 to 400 mbar, the λ increases proportionally as predicted by the expansion/compression of the ideal gas for ambient pressure ($P_0 \sim 1$ bar). However, we find the proportional coefficients, i.e., the slope, depends on frequencies; the slope decreases as the frequency increases, as shown in the inset. It is because the actual pressure-modulation amplitude experienced by bubbles at high frequencies is smaller than the set value ΔP, e.g., 400 mbar at 10 Hz. The finite response/settling time for our pressure controller to reach the set point pressure is greater than 50 ms; in fact, larger than $\sim$ 100 ms considering the volume of our pressure chamber. Namely, if the pulsation period becomes comparable with the response/settling time, the actual ΔP should be smaller than the set point.

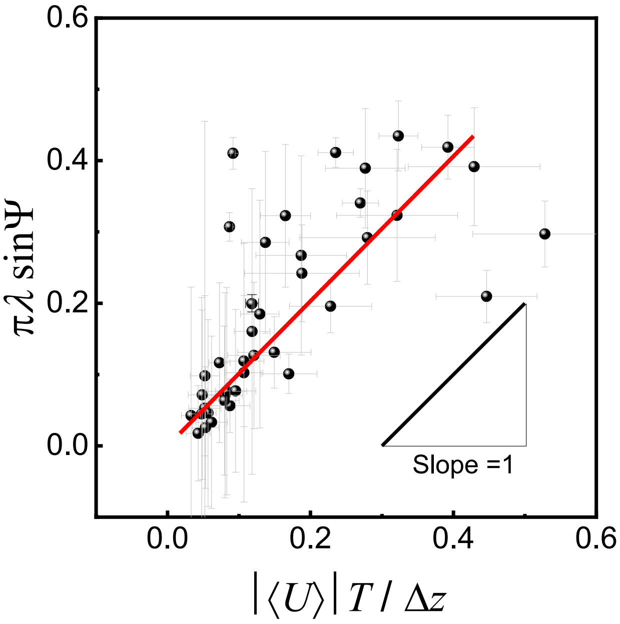

Supplementary Fig. 4. Another validation of our theoretical model. The first and third terms in square brackets in Eq. 6 represent the oscillation amplitude Δz and average swimming speed $\langle U\rangle$ of the bubble motion, respectively. Our model predicts that the ratio of one-cycle net displacement $|\langle U\rangle|T$ to the oscillation amplitude Δz should be equal to $\pi\lambda \sin\Psi$. Indeed, the experimental data show the proportional relation that the slope of the red linear fit line to the scattered data points is 0.99 ± 0.06. The error bars show standard deviations.

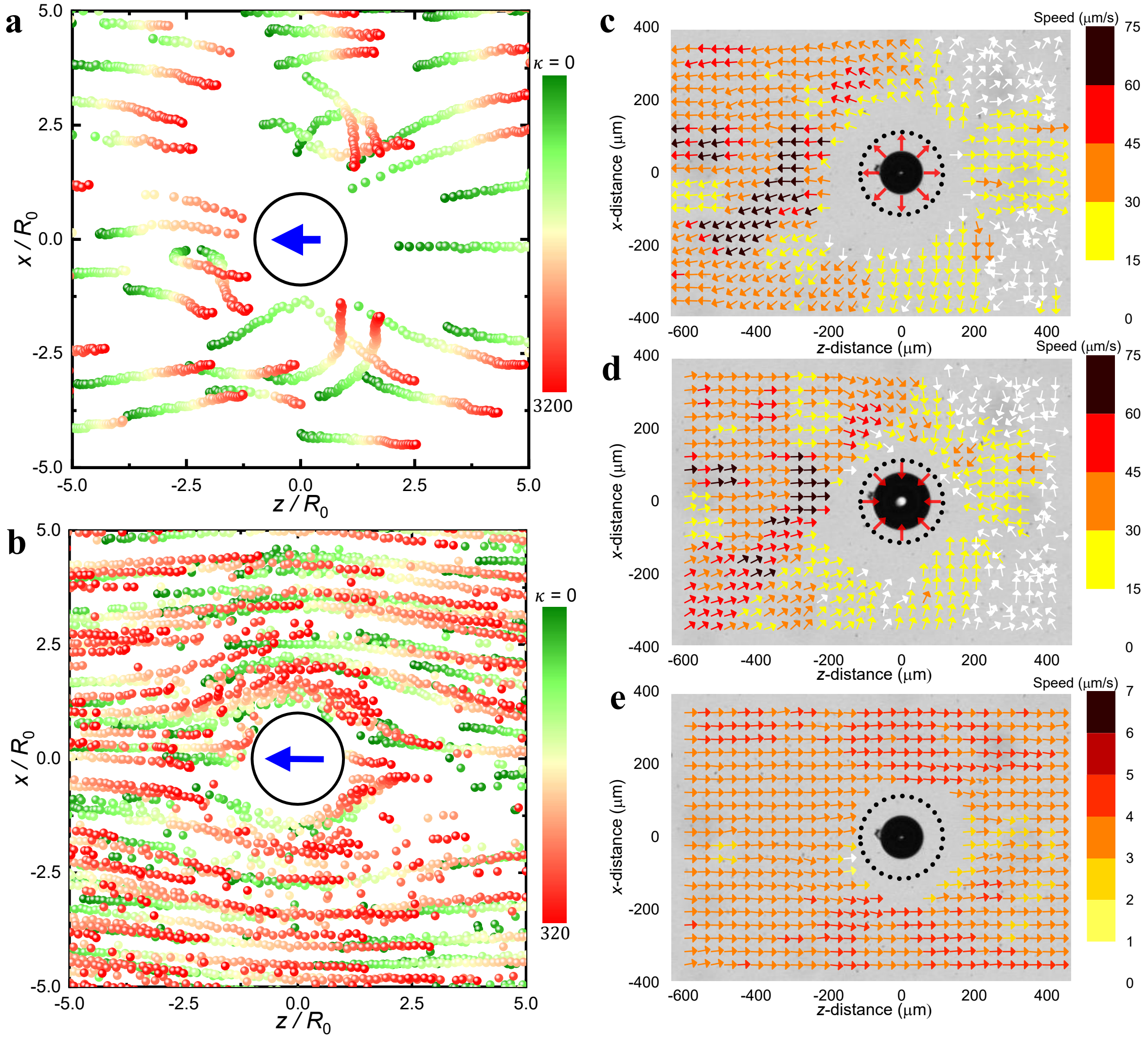

Supplementary Fig. 5. Flow fields around the pulsating bubble. **a**, Tracer trajectories around a representative pulsating HHB in the bubble's centre frame (Supplementary Movie 6). The black circle and blue arrow represent the HHB and its swimming direction, respectively. The stroboscopic trajectories of tracers with the colour representing the cycle number κ, show complex flow fields. Note that the trajectory is the xz projection of a microparticle's 3D motion with the cell thickness H = 155 μm and $\frac{2R_0}{H} \approx 0.7$. Stroboscopic trajectories (**b**) and particle image velocimetry (PIV) results (**c**–**e**) around a representative pulsating HHB at the bubble's centre frame when $\frac{2R_0}{H} \approx 1$ and H = 155 μm (Supplementary Movie 7). The trajectories in **b** are represented in the same way in **a**. The iterative PIV routine of ImageJ plugin with the normalized correlation coefficient algorithm (Template matching method) generated the PIV results from the microparticle trajectories: **c** during the expansion, **d** during the shrinkage, and time-averaged **e**. The direction and colour of the arrow represent the direction and speed of the flow-field vector, respectively. Note that the time-averaged flow vector profile shown in **e** looks similar to the stroboscopic result **b**, meaning that the PIV reproduces the fluid flow induced by the bubble propulsion.

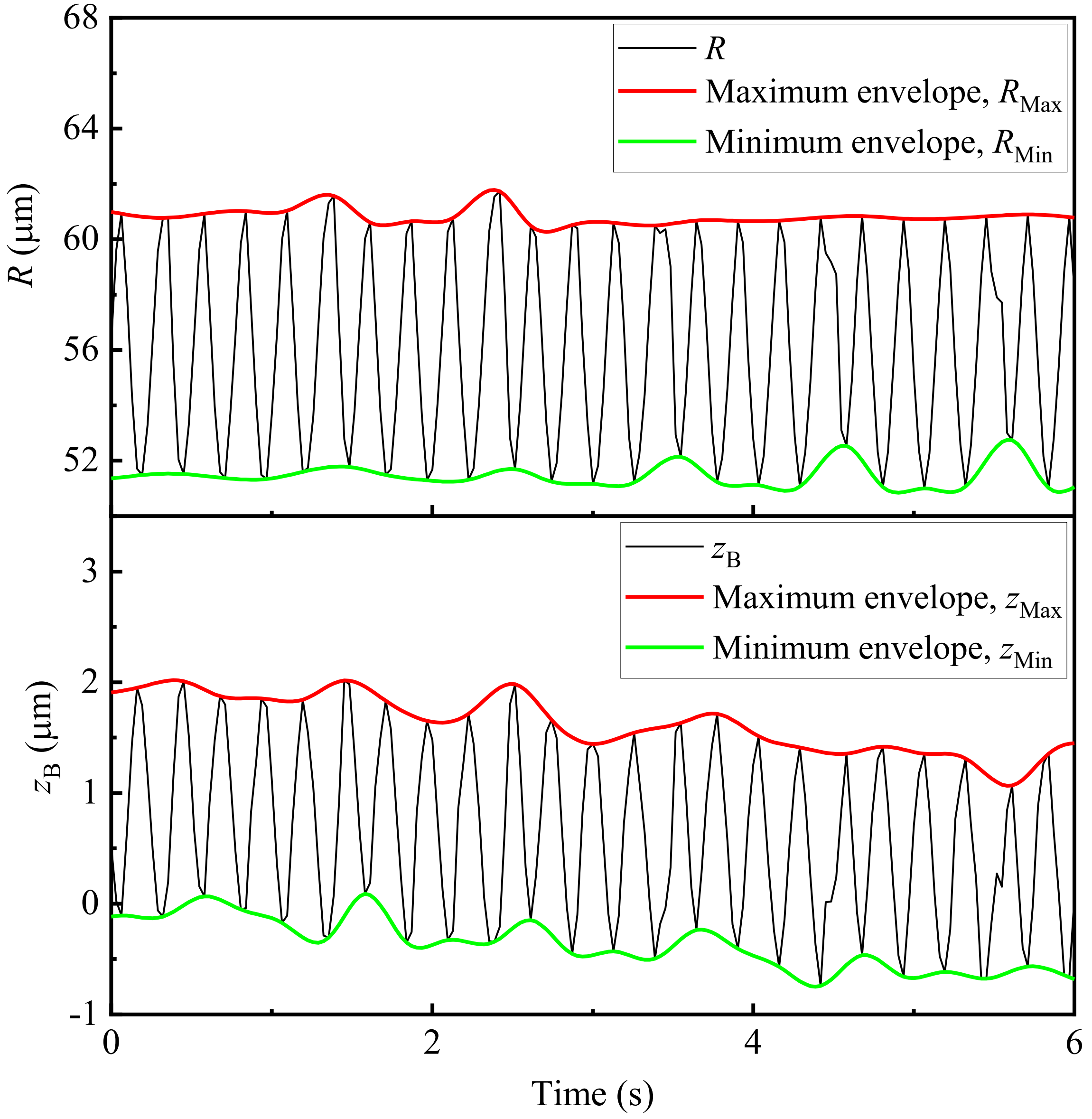

Supplementary Fig. 6. The envelop analysis of representative experimental data. The black solid lines show how the radius R and the centre position z_B of a pulsating HHB change over time. The red and green curves correspond to the maximum and minimum envelopes, respectively, from which we measure the oscillation amplitudes Δz and ΔR, and centre values such R_0 and z_0 as functions of time.
