# Supplementary material for: Symmetrically pulsating bubbles swim in an anisotropic fluid by nematodynamics
Source: Nat Commun. 2024 Feb 9;15:1220. doi: 10.1038/s41467-024-45597-1 (PMC10858235; doi:10.1038/s41467-024-45597-1)
Supplement: Supplementary file 10 — Description of Additional Supplementary Files [file 41467_2024_45597_MOESM10_ESM.pdf]

Title: Supplementary Movie 1.
Description: Shrinking process of bubbles. The positive offset pressure P_offset is applied without pulsation to decrease bubble sizes. The playback speed is $50\times$. The scale bar is 200~μm.

Title: Supplementary Movie 2.
Description: Motion of pulsating SRBs. The pulsating frequency of each movie increases from 0 (top) to 5 Hz (bottom) by 1 Hz. The playback speeds shown at the top-right corners change from $1\times$ to $10\times$. The scale bar is 100~μm.

Title: Supplementary Movie 3.
Description: Motion of pulsating HHBs. The pulsating frequency of each movie increases from 0 (top) to 5 Hz (bottom) by 1 Hz. The playback speeds at the top-right corners change from $1\times$ to $10\times$. The scale bar is 100~μm.

Title: Supplementary Movie 4.
Description: Swimming of multiple bubbles by pulsation. The playback speeds at the top-left corners change from $1\times$ to $100\times$. The scale bar is 200~μm.

Title: Supplementary Movie 5.
Description: Real-time observation of a pulsating HHB. The pulsating frequency is 1 Hz, and the scale bar is 100~μm.

Title: Supplementary Movie 6.
Description: Observation of tracer particles around a pulsating HHB for $\frac{2R_{0}}{H} \approx 0.7$. The pulsating frequency is 4 Hz, and the scale bar is 200~μm. The playback speed is $50\times$.

Title: Supplementary Movie 7.
Description: Observation of tracer particles around a pulsating HHB under strong confinement of $\frac{2R_0}{H} \approx 1$. The pulsating frequency is 4 Hz, and the scale bar is 200~μm. The playback speed is $10\times$.

Title: Supplementary Movie 8.
Description: Observation of a pulsating SRB with a bent SR (top) and a red guideline (bottom). The pulsating frequency is 4 Hz, and the scale bar is 200~μm. The playback speed shown at the top-left corner changes from $1\times$ to $10\times$.
